# Astaxanthin alleviates PM_2.5_-induced cardiomyocyte injury via inhibiting ferroptosis

**DOI:** 10.1186/s11658-023-00513-1

**Published:** 2023-11-25

**Authors:** Jingyi Ren, Bowen Yin, Zihao Guo, Xiaoya Sun, Huanting Pei, Rui Wen, Ziyi Wang, Siqi Zhu, Jinshi Zuo, Yadong Zhang, Yuxia Ma

**Affiliations:** 1 Department of Nutrition and Food Hygiene, School of Public Health, Hebei Key Laboratory of Environment and Human Health, Hebei Medical University, Shijiazhuang, 050017 China; 2 Undergraduate of College of Public Health, Hebei Medical University, Shijiazhuang, 050017 China

**Keywords:** Astaxanthin, Cardiomyocyte injury, Cardiovascular diseases, PM_2.5_, Ferroptosis

## Abstract

**Background:**

Long-term exposure of humans to air pollution is associated with an increasing risk of cardiovascular diseases (CVDs). Astaxanthin (AST), a naturally occurring red carotenoid pigment, was proved to have multiple health benefits. However, whether or not AST also exerts a protective effect on fine particulate matter (PM_2.5_)-induced cardiomyocyte damage and its underlying mechanisms remain unclear.

**Methods:**

In vitro experiments, the H9C2 cells were subjected to pretreatment with varying concentrations of AST, and then cardiomyocyte injury model induced by PM_2.5_ was established. The cell viability and the ferroptosis-related proteins expression were measured in different groups. In vivo experiments, the rats were pretreated with different concentrations of AST for 21 days. Subsequently, a rat model of myocardial PM_2.5_ injury was established by intratracheal instillation every other day for 1 week. The effects of AST on myocardial tissue injury caused by PM_2.5_ indicating by histological, serum, and protein analyses were examined.

**Results:**

AST significantly ameliorated PM_2.5_-induced myocardial tissue injury, inflammatory cell infiltration, the release of inflammatory factors, and cardiomyocyte H9C2 cell damage. Mechanistically, AST pretreatment increased the expression of SLC7A11, GPX4 and down-regulated the expression of TfR1, FTL and FTH1 in vitro and in vivo.

**Conclusions:**

Our study suggest that ferroptosis plays a significant role in the pathogenesis of cardiomyocyte injury induced by PM_2.5_. AST may serve as a potential therapeutic agent for mitigating cardiomyocyte injury caused by PM_2.5_ through the inhibition of ferroptosis.

**Supplementary Information:**

The online version contains supplementary material available at 10.1186/s11658-023-00513-1.

## Background

Globally, air pollution threatens human health and is a major contributor to morbidity and mortality [1]. According to recent World Health Organization (WHO) estimates, air pollution is estimated to be responsible for the deaths of 3.8 million people annually [2]. Fine particulate matter (PM_2.5)_ is widely acknowledged as a prominent risk factor that does great harm to human health amidst various air pollutants [3]. PM_2.5_ is defined as ambient particles with an aerodynamic diameter of less than or equal to 2.5 μm. It is a complex mixture of different components, including water-soluble components, polycyclic aromatic hydrocarbons (PAHs) and heavy metals [4]. The major sources of PM_2.5_ are automobile exhaust, road dust, coal combustion and industrial emissions [5]. The finer particle size of PM_2.5_ is able to penetrate the lungs and enter the blood circulation, thus causing harmful effects on respiratory and cardiovascular systems [6]. Over recent years, there has been an increasing awareness of the association between PM_2.5_ and adverse cardiovascular events. Epidemiological studies have shown that exposure to PM_2.5_ was highly correlated with diverse cardiovascular diseases (CVDs), such as heart failure, arrhythmia, hypertension and atherosclerosis [7]. Previous studies have confirmed that PM_2.5_ exposure aggravates inflammatory response and oxidative stress, which is the dominant mechanism of PM_2.5_-induced diseases [4, 8–11]. However, the above mechanism cannot fully explain PM_2.5-_induced cardiovascular injury, it is urgent to explore the potential molecular mechanisms to identify effective preventive and treatment strategies.

Ferroptosis is a novel form of regulatory cell death (RCD) triggered by iron-dependent lipid peroxidation, which is different from other RCD patterns, including autophagy, apoptosis, and necroptosis, in morphology and mechanism [12]. Several studies show compelling evidence that ferroptosis can be triggered by suppression of system Xc—activity, down-regulation of glutathione peroxidase 4 (GPX4), and overproduction of reactive oxygen species (ROS) [13, 14]. Accumulating evidence suggests that ferroptosis is related with the progression of various diseases, such as neurological disorders, respiratory diseases and CVDs [15, 16]. It may therefore be possible to treat cell death-related diseases by inhibiting ferroptosis. Ferrostatin-1 (Fer-1) is a potent inhibitor of ferroptosis. Studies have confirmed that Fer-1 can alleviate pulmonary and CVDs caused by multiple reasons [17–19]. A recent study demonstrated that Fer-1 could significantly reverse the toxicity of PM_2.5_ to human nasal epithelial cells [20]. Hu et al. [21] found that Fer-1 could reverse the promoting effect of ferritinophagy-mediated ferroptosis on PM_2.5_-induced cardiac fibrosis in mice. The above studies indicated that Fer-1 may provide a potential new therapeutic option for combating various diseases affected by air pollution.

Astaxanthin (AST), a naturally occurring lipid-soluble and red–orange carotenoid, is believed to possess advantageous properties for human health, including the mitigation of CVDs, various cancer types, and autoimmune diseases [22, 23]. These benefits are attributed to its potent anti-inflammatory and antioxidant characteristics [24, 25]. Nevertheless, the effect of AST on PM_2.5_-induced cardiac dysfunction remains not clear. Therefore, this study was performed to investigate whether AST inhibits ferroptosis and protects against PM_2.5_- induced cardiomyocyte injury in vivo and in vitro.

## Materials and methods

### Bioinformatics analysis

The RNA-seq dataset GSE211949 was downloaded from Gene Expression Omnibus (GEO, https://www.ncbi.nlm.nih.gov/geo/) database. This dataset which was performed on the GPL24247 comprised gene expression profiles from 3 normal mouse cardiomyocyte cells and 3 mouse cardiomyocyte cells after treatment with PM_2.5_ (100 μg/mL). Differentially expressed genes (DEGs) were selected with the “DEseq2” package implemented in R software (R version 4.0.3). Adjust *p* < 0.05 and |log2FoldChange|≥ 1 were determined as the screening condition. The DEGs of Kyoto Encyclopedia of Genes and Genomes (KEGG) analyses were conducted by using the “clusterProfiler” R package. The results with adjust *p* < 0.05 were considered significantly enriched and visualized by an online platform for data analysis and visualization (https://www.bioinformatics.com.cn). Ferroptosis-related genes (FRGs) were obtained from GeneCards [26] (https://www.genecards.org) and FerrDb [27] (https:// www.zhounan.org/ferrdb).

### Computational pharmacology prediction

The candidate targets of AST were predicted using TargetNet (http://targetnet.scbdd.com/), Similarity Ensemble Approach (SEA, http://sea.bkslab.org/), Swiss Target Prediction (https://swisstargetprediction.ch/), PharmMapper (http://lilab-ecust.cn/pharmmapper/index.html), Bioinformatics Analysis Tool for Molecular mechanism of Traditional Chinese Medicine (BATMAN-TCM, http://bionet.ncpsb.org.cn/batman-tcm/), and ChEMBL (https://www.ebi.ac.uk/chembl/). Subsequently, the targets were chosen by using the “VennDiagram” package (1.6.20) in R software and jvenn (https://jvenn.toulouse.inrae.fr/app/index.html) were inputted into the STRING database (https://string-db.org/) that was conducted to establish the PPI network. The built-in Degree algorithm of Cytohubba assigned a value to each target in the PPI network and ranked these genes by values. The top 10 targets were significant and were regarded as hub targets.

### Docking analysis

The 2D structure of AST was downloaded from PubChem (https://pubchem.ncbi.nlm.nih.gov/), and the A-fold structure of the hub target was gained from the UniProt database (https://www.uniprot.org/). The three-dimensional (3D) structure of the hub target was obtained from Protein Data Bank (PDB) database (http://www.rcsb.org/). PyMoL (version 1.7.2.1) software was used to shed excessive ligands. Docking ligands and receptors were inputted into AutoDock Tools V1.5.6 (http://autodock.scripps.edu/) software for routine pre-treatment and saved in PDBQT format. AutoDockVinaV1.1.2 was used to perform molecular docking. Finally, the 3D docking drawing was visualized by PyMOL software.

### Materials and reagents

AST was obtained from Solarbio Science & Technology Co., Ltd. (Beijing, China). Olive oil was obtained from yuanye Biotechnical Company (Shanghai, China). Fer-1 was obtained from MedChemExpress (Shanghai, China). The assay kits for creatine kinase (CK), lactate dehydrogenase (LDH), iron, superoxide dismutase (SOD), malondialdehyde (MDA), glutathione (GSH) and catalase (CAT) were purchased from Nanjing Jiancheng Bioengineering Institute (Nanjing, China). ELISA kits for interleukin-6 (IL-6), IL-1β and tumor necrosis factor-α (TNF-α) were purchased from Shanghai Enzyme-linked Biotechnology Co., Ltd (Shanghai, China). The primary antibodies against TfR1 (#A5865), FTL (#A11241), FTH1 (#A19544), GPX4 (#A1933), SLC7A11 (#A2413), GAPDH (#A19056) were obtained from ABclonal (Wuhan, China). Horseradish peroxidase-conjugated secondary antibody were obtained from Abways Technology (Shanghai, China). Detailed information regarding the antibodies is shown in the Additional file 1: Table S1.

### Collection of PM_2.5_ samples

The collection and preparation of PM_2.5_ samples were conducted according to our previously reported methods [4]. Briefly, PM_2.5_ was enriched on the quartz microfiber filters through a large-flow sampler. The quartz microfiber filters were sliced into small pieces soaked in the ultrapure water and sonicated with ultrasonic waves for 1 h. After filtering through the gauze, the suspension containing PM_2.5_ was dried with a vacuum freeze-dryer for 48 h to obtain the dry powder of PM_2.5_, and then PM_2.5_ was not merely split into equal aliquots but stored at − 80 °C.

### Cells cultures and treatments

The H9C2 rat myocardial cell line (ZQ0102) was supplied by Shanghai Zhong Qiao Xin Zhou Biotechnology Co., Ltd (Shanghai, China) and cultured in DMEM high-glucose medium supplemented with 10% FBS and 1% penicillin and streptomycin at 37 °C with 5% CO_2_. To choose the best concentration and intervention time, cells were exposed to different concentrations of PM_2.5,_ AST or Fer-1. Before treatment with PM_2.5_, cells were pretreatment with AST (20 μM, 40 μM), Fer-1 (1 μM) (MedChemExpress, China) for 1 h. Here, we would like to demonstrate that AST has a similar effect to ferroptosis inhibitor Fer-1.

### Cell viability assay

We used the Cell Counting Kit-8 (CCK-8; ZETA LIFE Inc., USA) to evaluate cell viability in our study. All procedures were performed according to instructions provided by the supplier. After cell grouping and treatment, the cells were washed with PBS. Absorbance was detected at 450 nm after the cells were incubated in a mixture of 10 μL CCK8 reagent and 100 μL fresh medium at 37 °C for another 2 h.

### Flow cytometric analysis of ROS

The H9C2 cells were pretreated with AST (20 and 40 μmol/L) for 1 h and then incubated with the PM_2.5_ for 24 h to establish cell injury model. Cells were incubated with the 5 μmol/L DCFH-DA (Nanjing Jiancheng Bioengineering Institute, Nanjing, China) working solution in a dark incubator at 37 °C for 30 min. Afterwards, the cells were rinsed with PBS two times and measured using flow cytometric analysis.

### Animal experiments

All experimental procedures were approved by the Ethics Review Committee for Animal Experimentation at Hebei Medical University (approval number: 2018025). All of the experiments were performed in accordance with the Helsinki declaration.

**Fig. 1:**
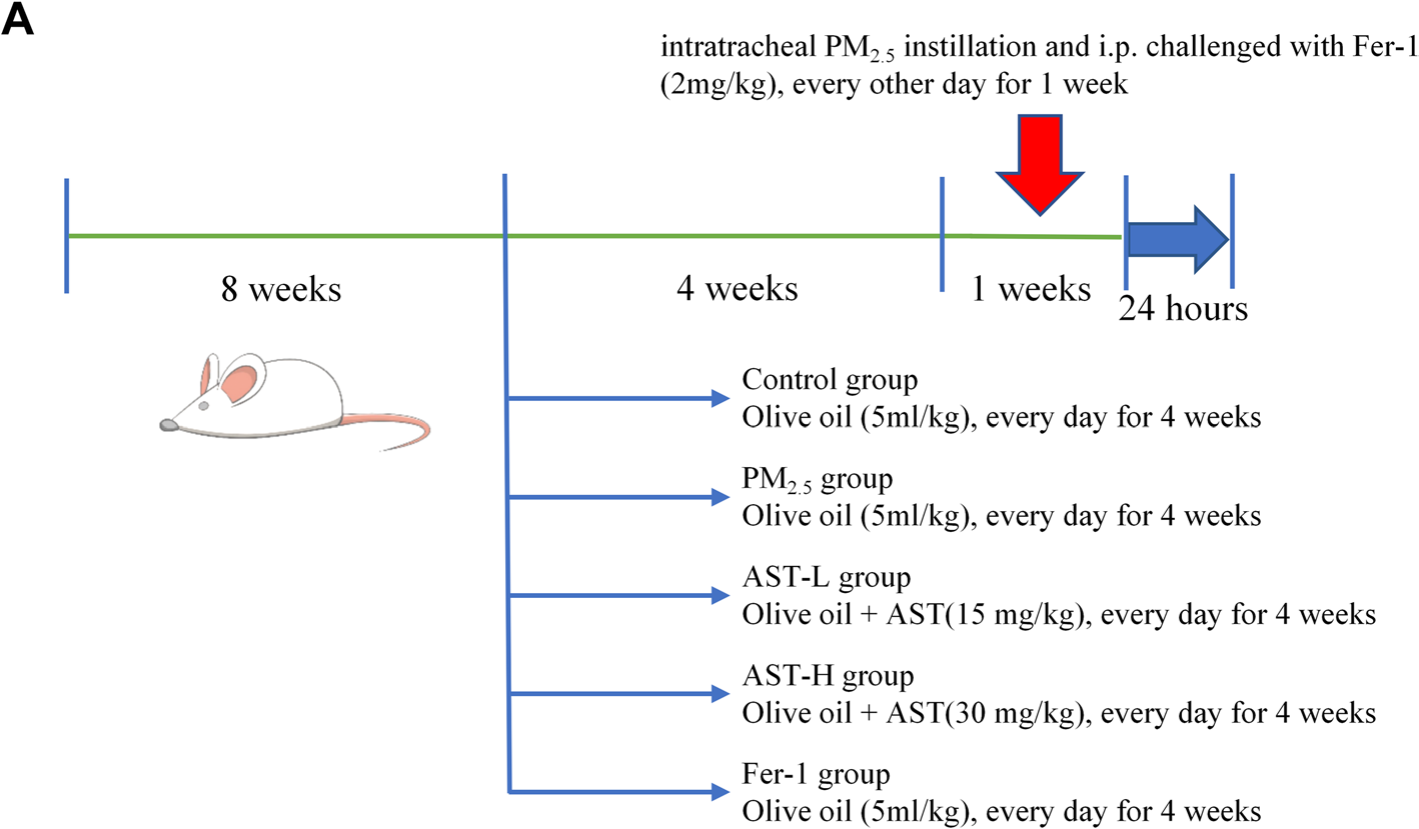
Schematic experimental design and dosage regimen in this study

Thirty male Sprague − Dawley rats (251 − 275 g) were gained from Beijing Vital River Laboratory Animal Technology Co., Ltd. The animals were acclimatized for 1 week under the standard environment (temperature, 20–24 °C; humidity, 50%-70%; photoperiod, 12:12 h). Afterwards, rats were randomly appointed into six groups (n = 6 for each group): (i) Control group, (ii) PM_2.5_ exposure group, (iii) Low-dose AST group (AST-L), (iv) High-dose AST group (AST-H), (v) Fer-1 group (Fer-1). The Control group, PM_2.5_ exposure group and Fer-1 group were given daily with olive oil (5 mL/kg/day) via oral gavage for 28 days. AST (AST-L:15 mg/kg; AST-H: 30 mg/kg) was dissolved in olive oil and was administered via gavage every day for 28 days. Intratracheal instillation began on the 22nd day, the SD rats were anesthetized with isoflurane and administered with sterile NaCl solution (1 mL per kg b.w.) or PM_2.5_ suspension (10 mg per kg b.w.) by intratracheal instillation every other day for a total of three times. The rats received intratracheal instillation of sterile NaCl solution in the control group every other day for 1 week. Ferroptosis inhibitor Fer-1 was administered intraperitoneally as a single dose of 2 mg/kg at 1 h before tracheal instillation in Fer-1 group. In the AST pretreatment groups and Fer-1 group, the rats were administered with PM_2.5_ suspension by intratracheal instillation every other day for 1 week. Twenty-four hours after the final exposure session, all of rats were executed after being anesthetized with pentobarbital sodium (50 mg/kg, i.p.), and then heart tissues and the serum samples of abdominal aorta were collected from the sacrificed rats. The specific study procedures is portrayed in Fig. 1.

### Histological analysis

As previously described [4], heart tissues were fixed in 4% paraformaldehyde overnight and embedded in paraffin. Hematoxylin–eosin (HE) was conducted to evaluate heart histological features. The severity of cardiac inflammation was evaluated using a previously described scoring system [28]. Superoxide anion production was evaluated using dihydroethidium (DHE) staining (Sigma-Aldrich). In brief, fresh frozen heart tissue sections were incubated with DHE (5 μM) away from light for 30 min at 37 °C. The cell nucleus were stained blue with Diaminophenyl indole (DAPI). Finally, the ROS level was evaluated by fluorescence microscope. All histological assessments were independently rated by two histologists who were blind to the procedure. Densitometric analysis and fluorescence intensity were carried out by using Image Pro Plus 6.0 software.

### Heart functional parameters

Isolated heart samples were washed with cold normal saline solution and a high-speed homogenizer was used to grind and homogenize the heart tissues for 5 min in 0.9% normal saline. Then, the homogenate was centrifuged at 3000 rpm for 10 min at 4 ℃ and the supernatant was collected. CK and LDH activity assay were used to assess heart function. The activities of CK and LDH were detected using the commercially available colorimetric assay kits (Nanjing Jiancheng Bioengineering Institute, Nanjing, China).

### Measurement of oxidative stress and inflammatory cytokines

Multiple oxidative stress markers (SOD, GSH, CAT, and MDA) were measured in serum and heart tissues using assay kits. The levels of inflammatory cytokines (IL-6, TNF-a, and IL-1β) in serum and heart tissues were detected by enzyme-linked immunosorbent assay according to manufacturer protocol.

### Detection of tissue iron

Myocardial iron content was detected by the commercially available colorimetric assay kits in strict accordance with the kit instructions (Nanjing Jiancheng Bioengineering Institute, Nanjing, China). Briefly, the optical density (OD) value was measured at 520 nm using a spectrophotometer and the iron content in the samples was then determined by comparing the OD of the samples to the standard curve.

### Western blot

Cells or tissue were lysed with the mixture of RIPA buffer and protease inhibitors, and centrifuged at 4 °C and 12,000 rpm to extract protein supernatant. The supernatant was subsequently denatured by boiling in 5 × (SDS) loading buffer for 5 min. Samples were separated by 8–12% (SDS-PAGE) and then electrically transferred onto (PVDF) membranes. The membranes were blocked in 5% skim milk for 2 h, then cut into strips and incubated with primary antibody overnight at 4 °C overnight. The next day, blots were washed four times with TBST for 5 min each time and incubated for 1 h with the corresponding secondary at room temperature. Subsequently, the bands were washed again with TBST four times. The bands were visualized with an enhanced chemiluminescence detection system. The gray density were finally quantified using Image J software. Expression levels were normalized using GAPDH as an internal control.

### Statistical analysis

The SPSS 21.0 software and the GraphPad Prism software were served as Statistical analysis. Numerical data were expressed as the mean ± standard error of the mean (SEM) and compared by one-way analysis of variance (ANOVA). *p* < 0.05 indicated statistical significance.

## Result

### Network pharmacology prediction

**Fig. 2:**
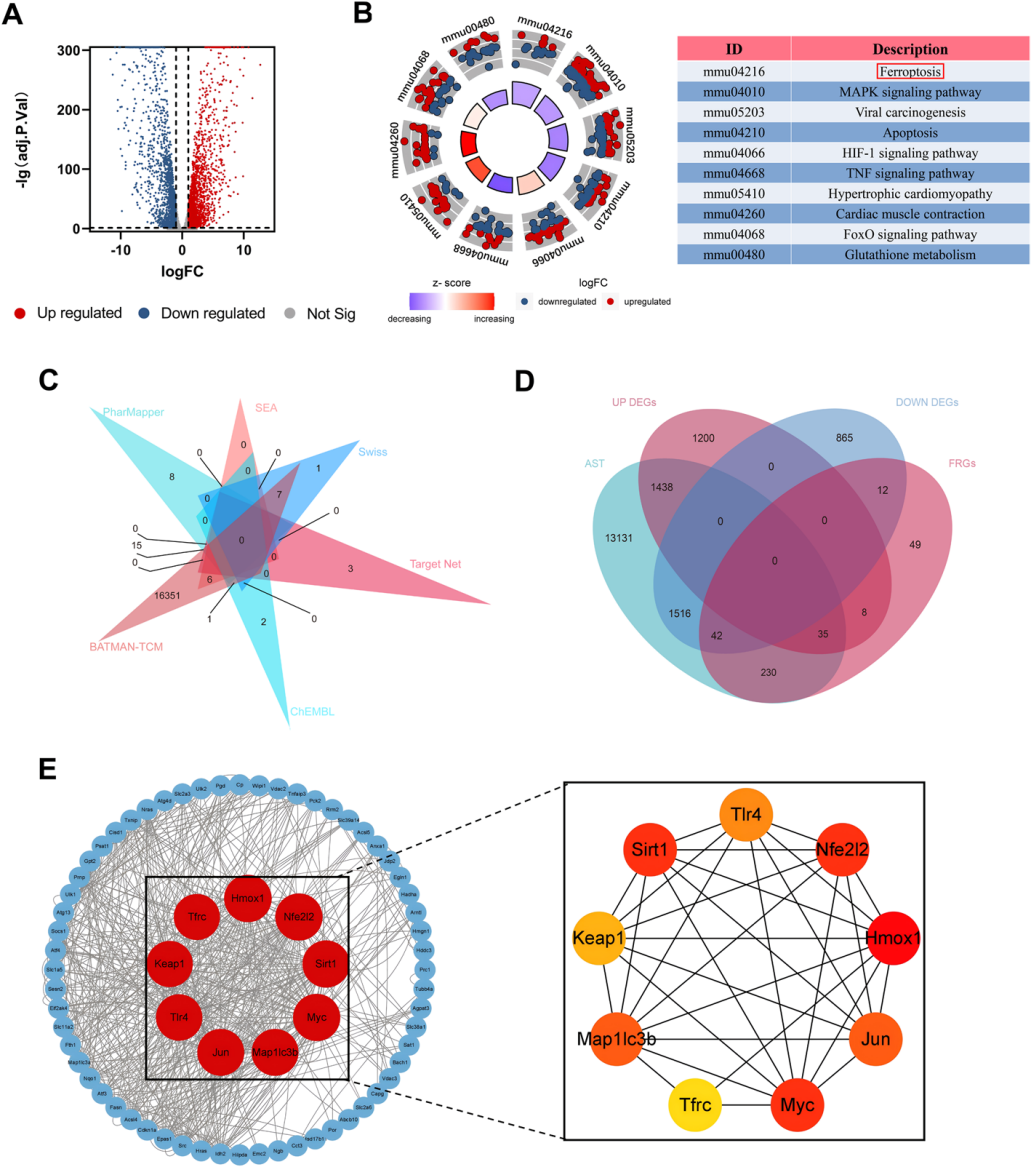
Computational pharmacology analysis of PM_2.5_ and astaxanthin. **A** The DEGs between normal mouse cardiomyocyte cells and cardiomyocyte cells after treatment with PM_2.5_. **B** KEGG enrichment analysis of DEGs. **C** Venn graph showing the numbers of predicted AST targets. **D** The overlapping targets of AST, FRGs, and DEGs. **E** The PPI network of overlapping targets. The red nodes represent the core targets

There were 5116 DEGs in the mouse cardiomyocytes treated with PM_2.5_ compared with the normal mouse cardiomyocytes (Fig. 2A). As showcased in Fig. 2B, KEGG pathway enrichment analyses were conducted to identify the biological function of the DEGs. The results suggested that the DEGs were majorly involved in Ferroptosis, MAPK signaling pathway, Viral carcinogenesis, Apoptosis, HIF-1 signaling pathway, TNF signaling pathway, Hypertrophic cardiomyopathy, Cardiac muscle contraction, FoxO signaling pathway, and Glutathione metabolism. From SEA, Swiss Target Prediction, PharmMapper, BATMAN-TCM, TargetNet, and ChEMBL databases, we obtained 16,392 target genes of AST after removal of the partially overlapped targets (Fig. 2C). 376 genes searched by GeneCards with “ferroptosis” as the keyword and FerrDb were taken into account as FRGs. As shown in Fig. 2D, there were 77 target genes overlapped among the target genes of AST, FRGs, and DEGs. A PPI network was established to search the interactions of these target genes through the STRING database. Cytoscape was used to further visualize the data. As showcased in Fig. 2E, there are multiple associations between hub genes and other target genes. The top 10 hub genes ranked by the Degree algorithm were then identified using the Cytohubba plugin, including Hmox1, Myc, Nfe2l2, Sirt1, Jun, Map1lc3b, Tlr4, Keap1, and Tfrc. Molecular Docking Analysis was used to confirm whether the bioactive components of AST could directly interact with the hub genes. As showed in Table 1, AST and the hub genes exhibited very good binding activity. The binding affinity lower than − 5.0 kcal/mol indicates that the active ingredient has a good binding ability to protein. Above all, by the effective combination of the bioactive compounds of AST and hub genes, we predicted that AST could alleviate the ferroptosis caused by PM_2.5_.

**Table 1:** The docking result analysis of AST and hub targets

Target protein	Binding energy (kcal/mol)	No of H bonds	Amino acid residues forming H-bond with their length in A
Hmox1	− 7.2	1	SER-159 (3.4)
Myc	− 7.7	2	GLU-383 (3.2), LYS-398 (2.6)
Nfe212	− 6.7	1	GLY-81 (2.5)
Sirt1	− 7.2	1	LYS-183 (2.2)
Jun	− 6.0	2	SER-174 (3.5), PHE-176 (3.4)
Map1lc3b	− 7.5	1	ASN-236 (2.8)
Tlr4	− 7.8	3	THR-497 (3.0), SER-332 (3.1), GLN-331 (2.6)
Keap1	− 9.5	1	VAL-463 (3.3)
Tfrc	− 8.2	1	PHE-299 (2.1)

### AST protects against PM_2.5_-induced cell injury in H9C2 cells

**Fig. 3:**
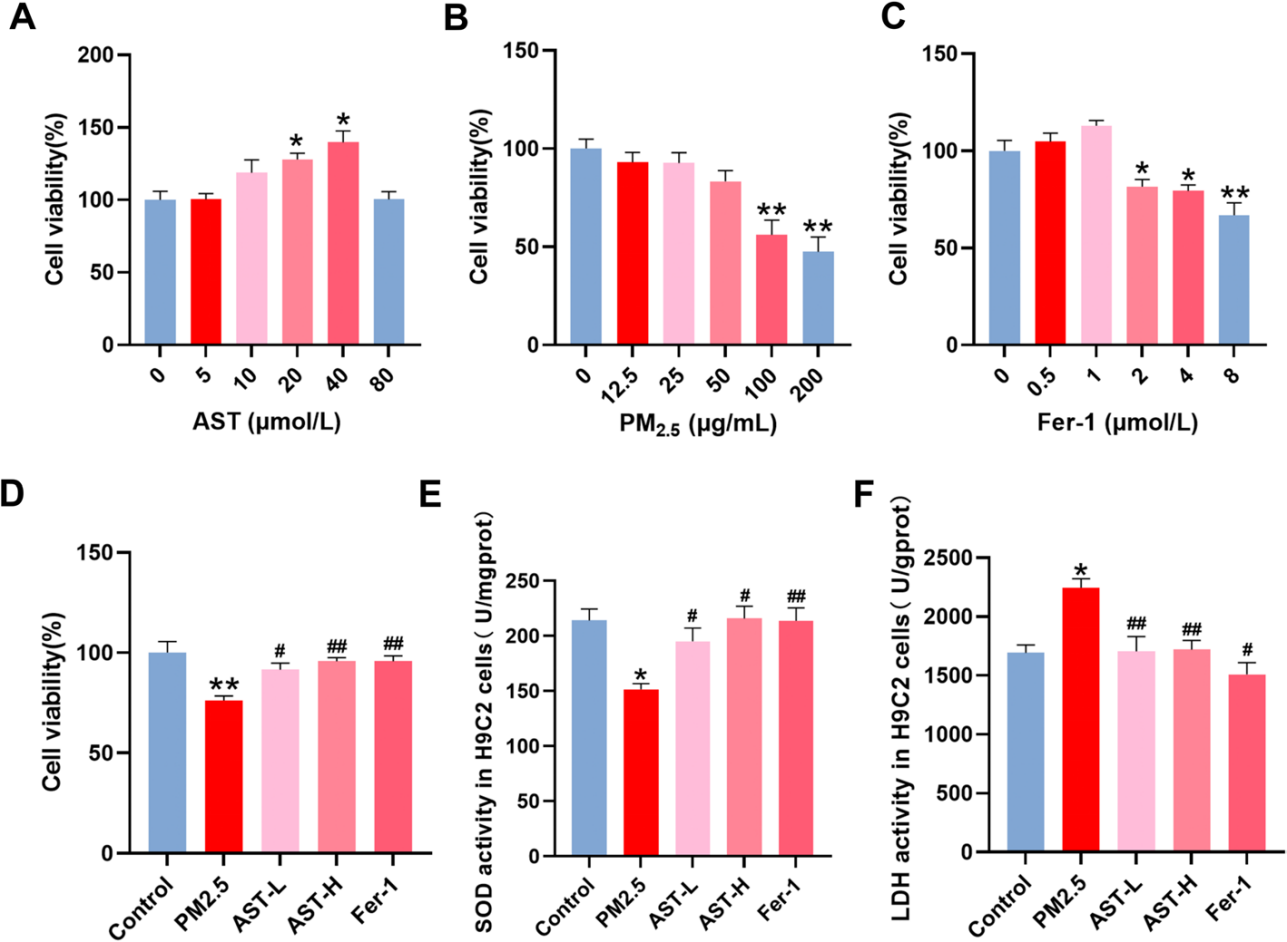
Astaxanthin attenuates PM_2.5_-induced H9C2 cells injury. H9C2 cells were treated with different concentrations of AST (**A**), PM_2.5_ (**B**), and Fer-1 (**C**) for 24 h. The cell viability was detected by CCK8 assay. **D** Cell viability of H9C2 cells treated with PM_2.5_, AST and Fer-1. The activities of SOD (**E**) and LDH (**F**) in the H9C2 cells treated with PM_2.5_, AST and Fer-1. Values are expressed as mean ± SEM (n = 6). *p < 0.05 difference from the control group; **p < 0.001 difference from the control group; ^*#*^*p* < 0.05 difference from the PM_2.5_ exposure group; and ^*##*^*p* < 0.001 difference from the PM_2.5_ exposure group

To determine the optimal concentration of AST, PM_2.5_, and Fer-1, H9C2 cells were treated with different concentrations of AST (0–80 µM), PM_2.5_ (0–200 µg/mL) or Fer-1 (0–8 µM) for 24 h (Fig. 3A–C). The cell viability was detected by CCK8 assay. From these preliminary findings, we selected the optimal concentrations of AST (20 µM, 40 µM), PM_2.5_ (100 µg/mL), and Fer-1 (1 µM). To evaluate the protective effects of AST against cell injury caused by PM_2.5_ in H9C2 cells, cells were pretreated with AST and fer-1 for 1 h and then exposed to PM_2.5_ for 24 h. We found that pretreatment with AST and fer-1 could significantly protect the proliferative ability (Fig. 3D), increase the activities of SOD (Fig. 3E), and inhibit LDH release (Fig. 3F). Above results indicate that the appropriate dose of AST effectively attenuates PM_2.5_-induced cell injury.

### AST attenuates PM_2.5_-induced myocardial injury in rats

**Fig. 4:**
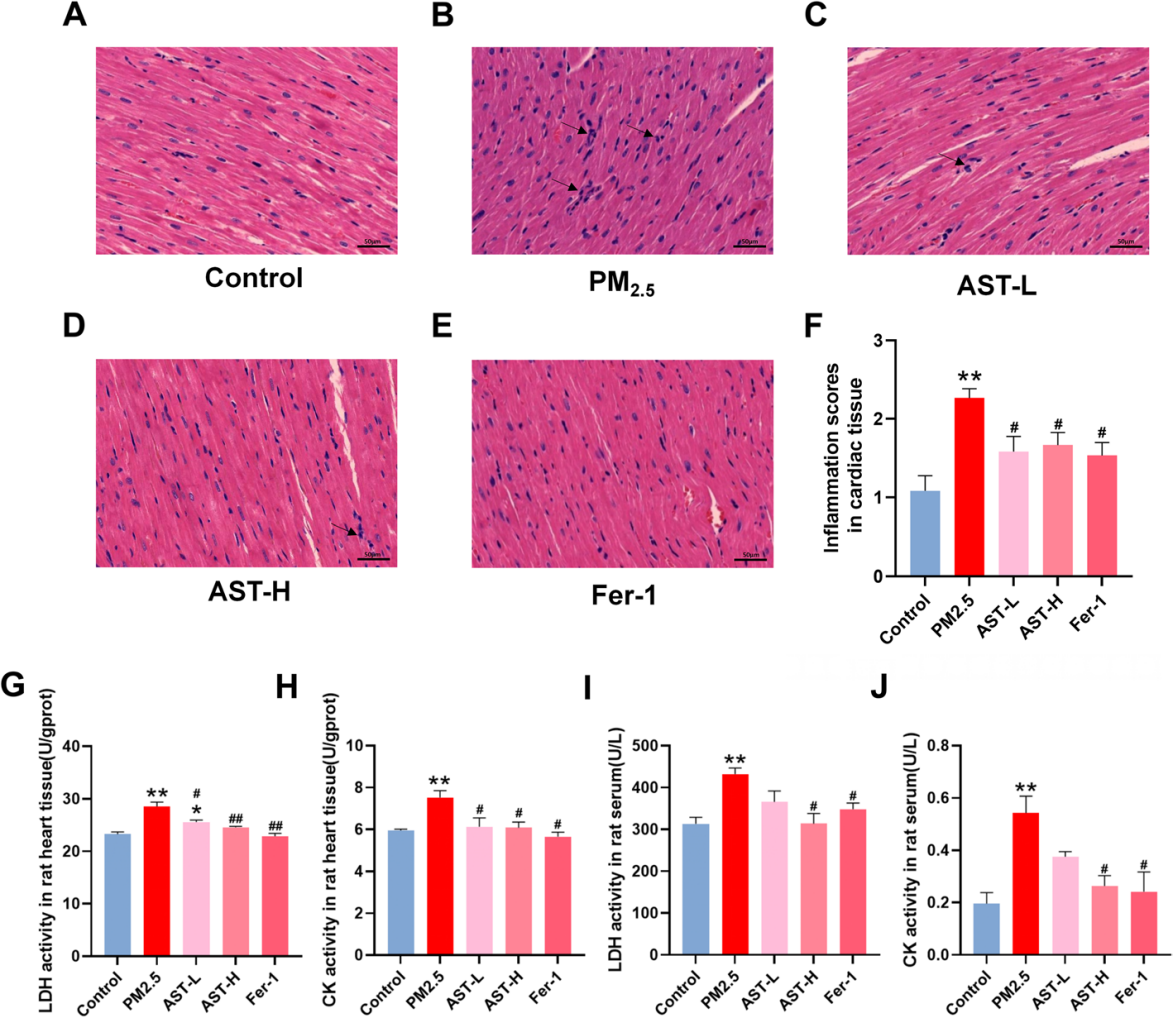
Astaxanthin alleviates heart injury induced by PM_2.5_ in mice. **A**–**E** Hematoxylin and eosin (HE) staining of heart tissue sections from different groups (Scale bar = 50 μm). **F** The myocarditis score of heart in each group. The activities of Lactate dehydrogenase (LDH) and creatine kinase (CK) activity in heart heart tissue (**G**, **H**) and serum (**I**, **J**) were measured. Values are expressed as mean ± SEM (n = 6). *p < 0.05 difference from the control group; **p < 0.001 difference from the control group; ^*#*^*p* < 0.05 difference from the PM_2.5_ exposure group; and ^*##*^*p* < 0.001 difference from the PM_2.5_ exposure group

To further determine the effect of AST on myocardial injury caused by PM_2.5_, in vivo experiments were performed using male SD rats. HE staining showed that AST and fer-1 could attenuate tissue edema, hemorrhage, and inflammatory cell infiltration induced by PM_2.5_ (Fig. 4A–E). To determine the cardioprotective effect of AST, the histopathological scores of heart tissues was conducted. The results indicated that exposure to PM_2.5_ notably elevated but AST and fer-1 pretreatment markedly reduced the histopathological scores of heart tissues (Fig. 4F). Similarly, AST and fer-1 significantly prevented PM_2.5_-induced cardiac dysfunction, decreased the activities of CK and LDH in heart tissues and serum (Fig. 4G–J).

### AST relieves PM_2.5_-induced myocardial inflammation in rats

**Fig. 5:**
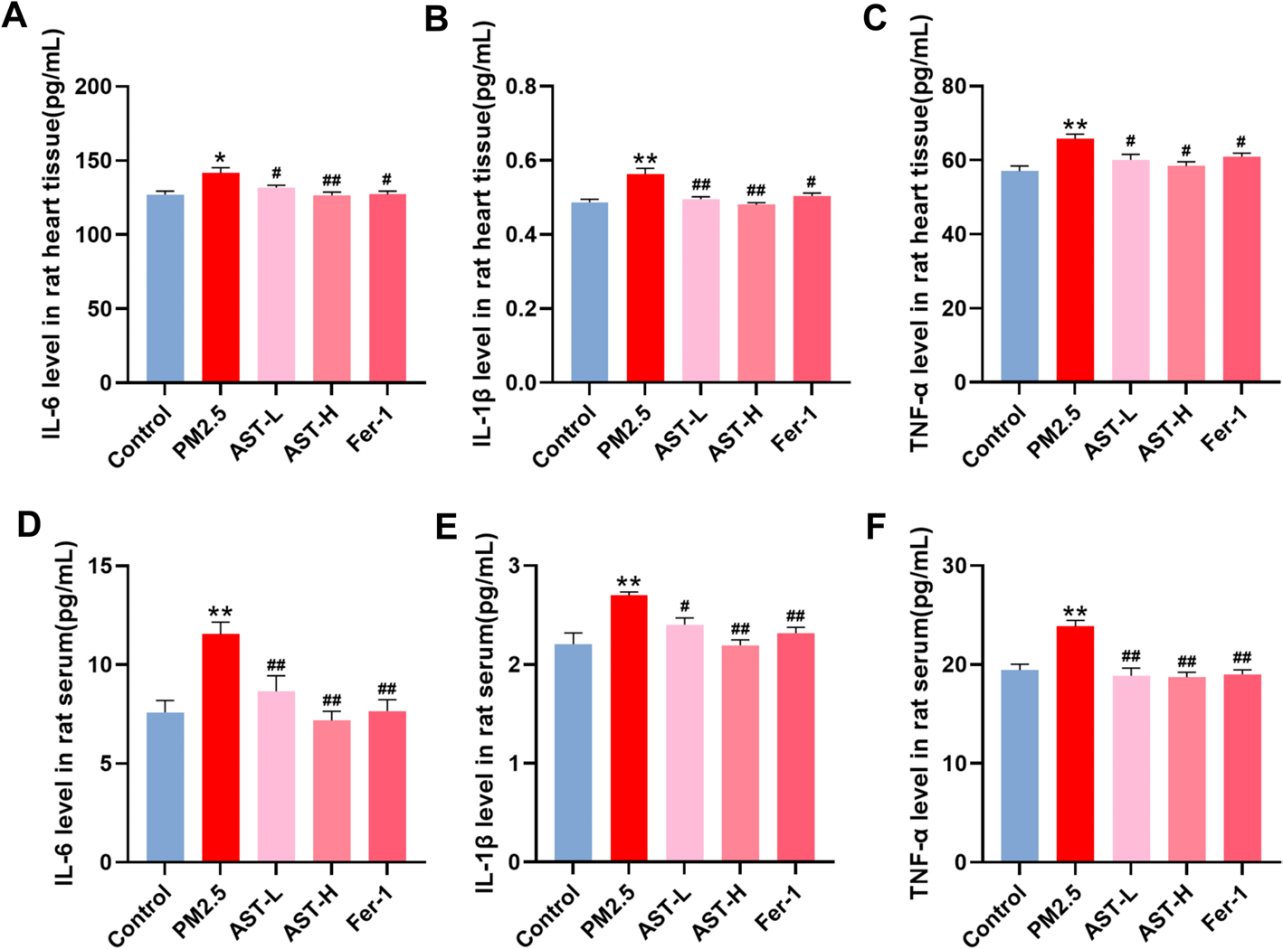
Effect of astaxanthin on the levels of IL-6, IL-1β and TNF-α in serum and heart. ELISA for TNF-α, IL-1β and IL-6 in heart tissue (**A**–**C**) and serum (**D**–**F**). Values are expressed as mean ± SEM (n = 6). *p < 0.05 difference from control group; **p < 0.001 difference from the control group; ^*#*^*p* < 0.05 difference from the PM_2.5_ exposure group; and ^*##*^*p* < 0.001 difference from the PM_2.5_ exposure group

In order to demonstrate the effect of AST in inflammatory response, the level of IL-6, IL-1β, and TNF-a in heart tissues and serum were detected. As shown in Fig. 5A–F, the levels of IL-6, IL-1β, and TNF-a in the heart tissues and serum were significantly increased in the PM_2.5_ exposure group, however, this up-regulation was relieved by AST and Fer-1. Thus, pretreatment with AST and Fer-1 largely suppressed the inflammatory response caused by PM_2.5_.

### AST attenuates PM_2.5_-induced myocardial oxidative stress in rats

**Fig. 6:**
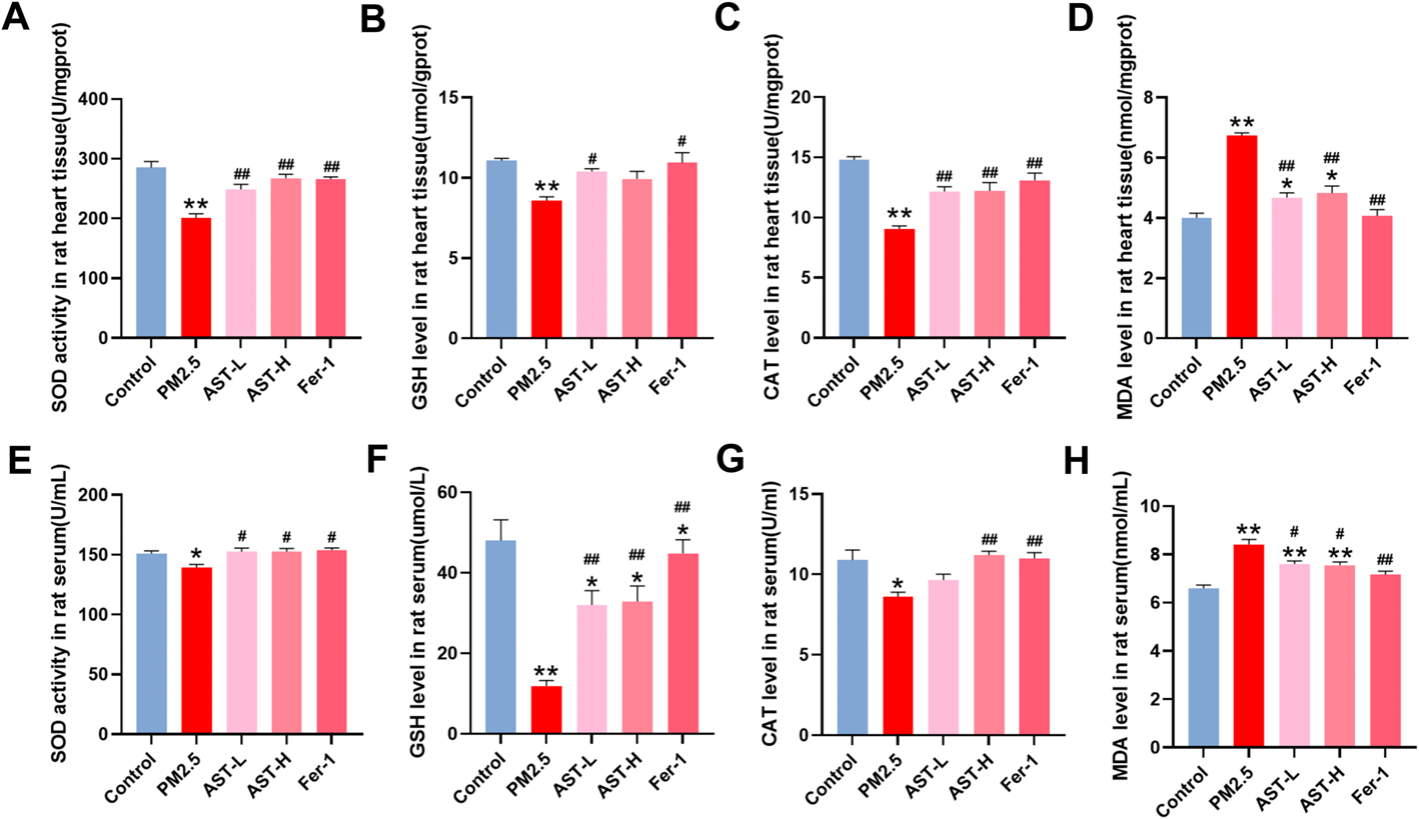
Effect of astaxanthin on the levels of SOD, CAT, GSH and MDA in serum and heart. The activities of SOD, GSH, and CAT in the heart tissue (**A**–**C**) and serum (**E**–**G**) were detected. Levels of MDA was measured in the heart tissue (**D**) and serum (**H**). Values are expressed as mean ± SEM (n = 6). *p < 0.05 difference from control group; **p < 0.001 difference from the control group; ^*#*^*p* < 0.05 difference from the PM_2.5_ exposure group; and ^*##*^*p* < 0.001 difference from the PM_2.5_ exposure group

Oxidative stress activation plays an essential role in the development of myocardial injury induced by PM_2.5_. Here, we detected MDA contents and the activities of typical antioxidant enzymes activities (SOD, GSH, CAT) in the heart tissues and serum (Fig. 6). Our data indicated that the activities of SOD, GSH, and CAT substantially decreased in the PM_2.5_ exposure group compared with that in the control group, which could be suppressed by AST and Fer-1 pretreatment in the heart tissues and serum. In addition, MDA content was evident as an increase in the PM_2.5_ exposure group, whereas AST pretreatment and Fer-1 significantly decreased the contents of MDA. Taken together, these data showed that AST exerted antioxidant effects in PM_2.5_-induced cardiomyocyte injury.

### AST inhibits PM_2.5_-induced ferroptosis in vitro

**Fig. 7:**
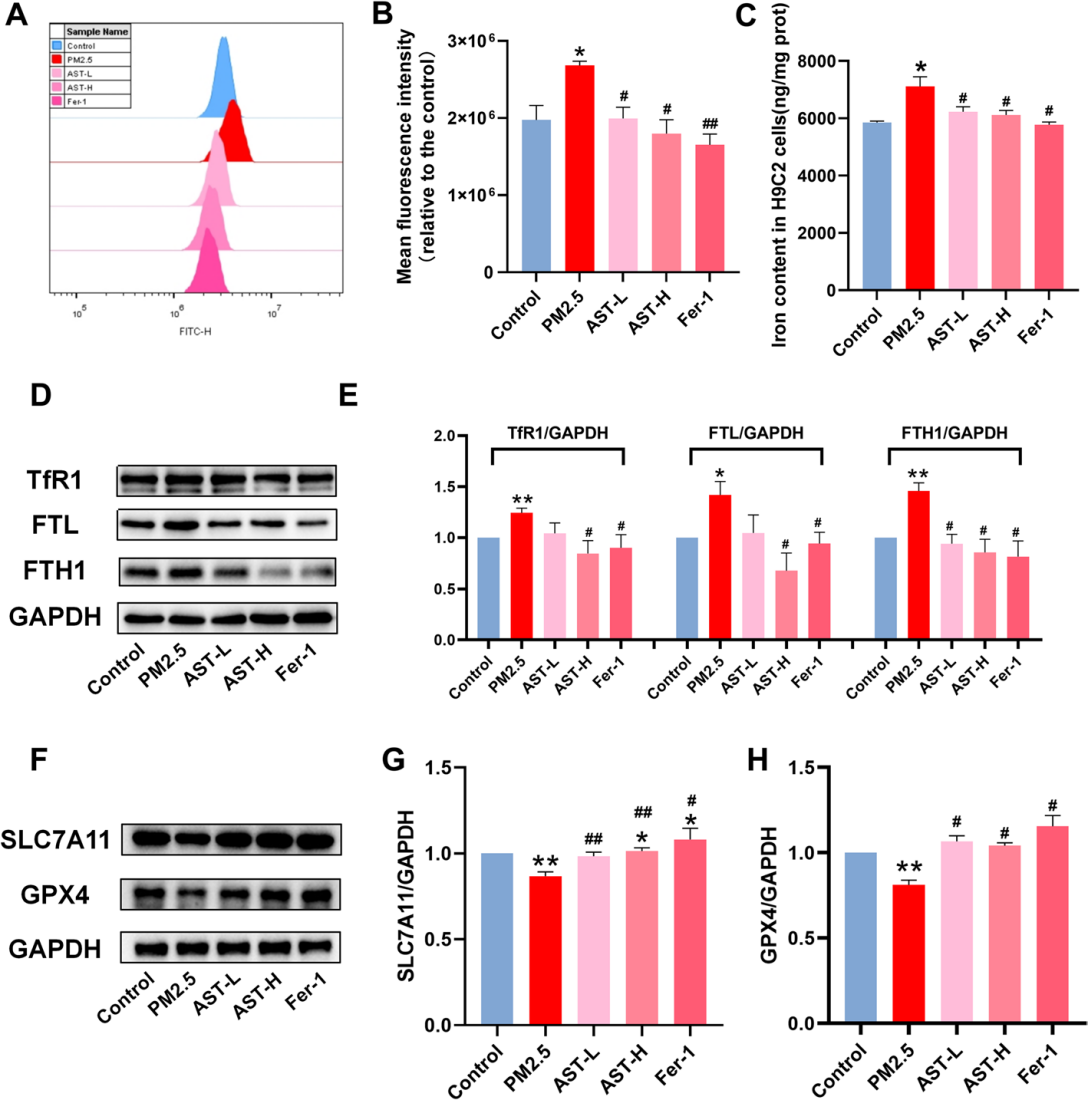
Astaxanthin attenuates PM_2.5_-induced ferroptosis in H9C2 cells. **A** Representative fluorescence intensity images of ROS by DCFH-DA obtained by flow cytometry. **B** Flow cytometric analysis of fluorescence intensity. **C** Iron content were detected in the H9C2 cells. **D**–**H** Western blot bands showing TfR1, FTL, FTH1, GPX4 and SLC7A11 protein expression and the relative signal intensities in H9C2 cells. For quantification, the intensity was normalized to GAPDH and the control was set to 1. Values are expressed as mean ± SEM (n = 6). *p < 0.05 difference from control group; **p < 0.001 difference from the control group; ^*#*^*p* < 0.05 difference from the PM_2.5_ exposure group; and ^*##*^*p* < 0.001 difference from the PM_2.5_ exposure group

To further confirm whether the protective effect of AST on PM_2.5_-induced myocardial injury is involved in inhibiting ferroptosis, we assessed indices of ferroptosis, including iron and ROS accumulation, and the expression levels of iron metabolism-related protein (TfR1, FTH1, and FTL) and ferroptosis-related proteins (GPX4 and SLC7A11). Our results showed that treatment with PM_2.5_ exposure obviously increased intracellular ROS and the concentration of iron in H9C2 cells (Fig. 7A–C). Similar to the anti-ferroptosis effect of Fer-1 and AST also obviously inhibits excessive accumulation iron and intracellular ROS overproduction (Fig. 7A–C). Transferrin receptor 1 (TfR1), a cell surface receptor that is responsible for transferrin-mediated iron uptake and is required for cellular iron uptake. We found that PM_2.5_ exposure increased the expression of TfR1 protein, whereas pretreatment with AST and Fer-1 restored its expression (Fig. 7D, E). The protein expression levels of ferritin heavy chain (FTH1) and ferritin light chain (FTL), were upregulated by PM_2.5_ exposure but AST pretreatment reduced its expression (Fig. 7D, E). In addition, the expression levels of ferroptosis-related proteins solute carrier family 7 member 11 (SLC7A11) and glutathione peroxidase 4 (GPX4) substantially decreased in the PM_2.5_ exposure group compared with that in the control group. However, this downregulation was inhibited by AST and Fer-1 (Fig. 7F–H).

### AST suppresses PM_2.5_-induced ferroptosis in vivo

**Fig. 8:**
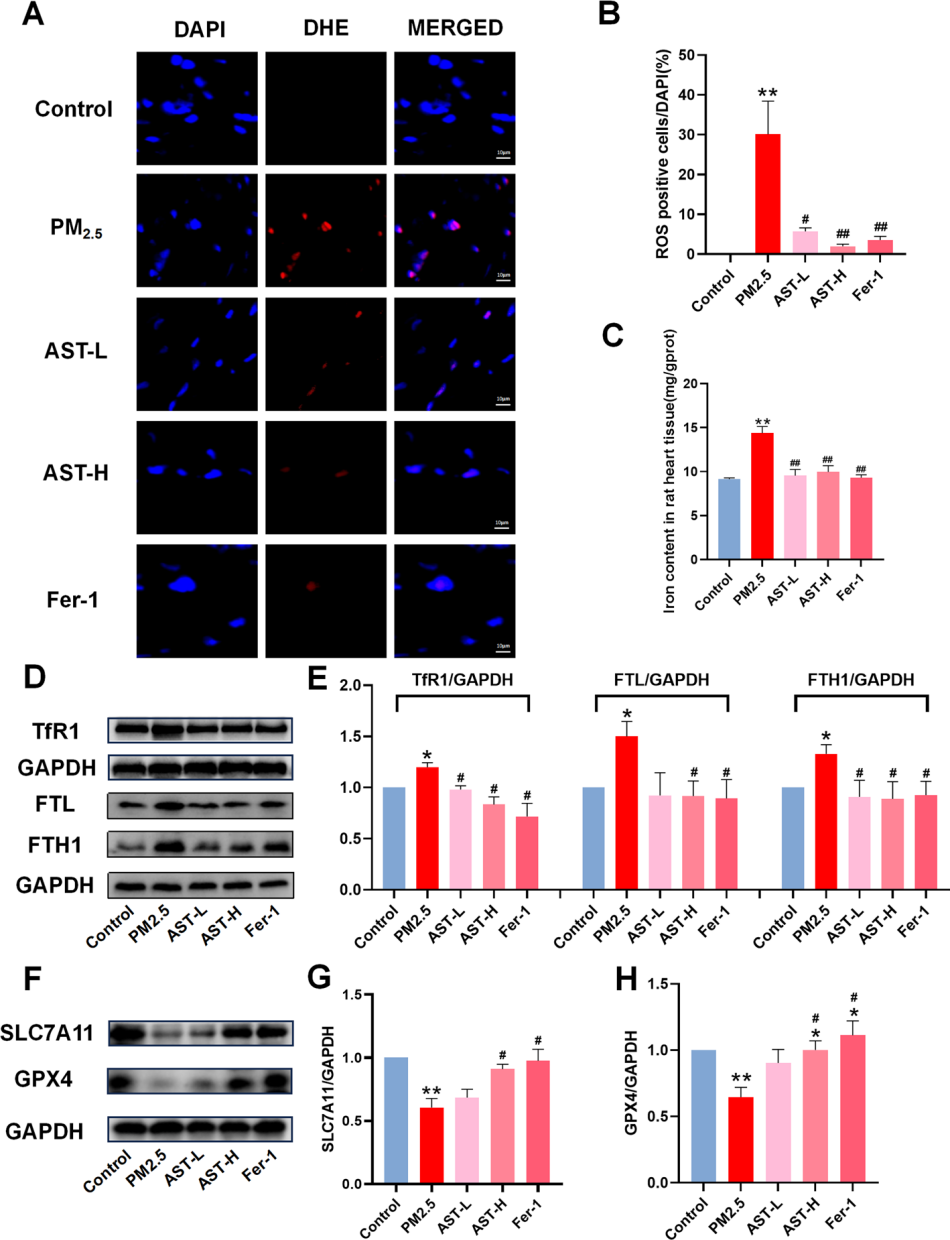
Astaxanthin attenuates PM_2.5_-induced ferroptosis in heart. Representative images of fluorescence probe for ROS and its statistical results in heart tissue (**A**, **B**). Iron content were detected in the heart tissues (**C**). Western blots for TfR1, FTL, FTH1, GPX4, and SLC7A11 in heart tissue (**D**–**H**). For quantification, protein expression was normalized to GAPDH and the control was set to 1. Values are expressed as mean ± SEM (n = 6). *p < 0.05 difference from the control group; **p < 0.001 difference from the control group; ^*#*^*p* < 0.05 difference from the PM_2.5_ exposure group; and ^*##*^*p* < 0.001 difference from the PM_2.5_ exposure group

The results in vivo were consistent with those in cells, the levels of iron and ROS were remarkably elevated in the PM_2.5_ exposure group compared to other groups. AST and Fer-1 substantially relieved PM_2.5_-induced changes in these indicators of ferroptosis (Fig. 8A–C). The ferroptosis level was evaluated by detecting the expression levels of iron metabolism-related proteins (TfR1, FTH1, and FTL) and ferroptosis-related proteins (SLC7A11 and GPX4) in the heart tissues using western blot. Compared with the control group, the expression of TfR1, FTH1and FTL in PM_2.5_ exposure group was remarkably increased. AST and Fer-1 obviously decreased the expression of TfR1, FTH1 and FTL (Fig. 8D, E). The expression level of GPX4 and SLC7A11 in the PM_2.5_ group was considerably decreased. By contrast, AST and Fer-1 pretreatment obviously suppressed the downregulation of ferroptosis-related proteins (Fig. 8F–H).

## Discussion

Epidemiological studies have suggested that long-term PM_2.5_ exposure is related to an increased risk of CVDs [29, 30]. However, there are no effective practical strategies to prevent or treat PM_2.5_-induced cardiovascular injury. Previous research propose that overproduction of ROS and excessive inflammatory factor release (e.g. IL-6 and TNF-a) plays a vital role in the pathogenesis of CVDs, and cell death is also recognized as a key mechanism of cardiovascular injury induced by PM_2.5_ [31, 32]. Apoptosis and autophagy have long been regarded as the main form of cell death for PM_2.5_-induced cell damage [33, 34]. However, due to excessive ROS accumulation exists in the PM_2.5_-induced cardiovascular injury, other forms of cell death may also play a more important role in CVDs caused by PM_2.5_. Recently, some studies have illustrated that ferroptosis due to the imbalance of iron metabolism are closely related to cardiomyocyte injury caused by PM_2.5_ [4, 21]. Thus, blocking ferroptosis may be a potential prevention and control strategy. In this study, the data showed that AST significantly attenuated oxidative stress and inflammatory damage due to PM_2.5_ exposure in vivo and in vitro. In addition, ferroptosis inhibitor Fer-1 was used to further investigate the underlying mechanism. The study results indicated that AST pretreatment could markedly protect cardiomyocytes from PM_2.5_-induced ferroptosis in vitro and in vivo. This is consistent with the function of Fer-1 against ferroptosis.

Network pharmacology provides a new research method for studying disease signaling disturbances and drug modes of action. Study on the Potential Molecular Mechanism of drug therapy based on network pharmacology and bioinformatic analysis, which offer a novel perspective for drug discovery and study of disease mechanisms. Through information biology analysis, 5116 DEGs were obtained from the database of GEO, which regulated multiple signaling pathways and played a role in treating cardiovascular injury induced by PM_2.5_. In KEGG enrichment analysis, the targets of PM_2.5_ were considerably enriched to various biological processes, including Ferroptosis, TNF signaling pathway, Hypertrophic cardiomyopathy, Cardiac muscle contraction, Glutathione metabolism, and so forth. The results showed that PM_2.5_-induced myocardial damage may be linked to ferroptosis. Multiple drug target databases (SEA, TargetNet, Swiss Target Prediction, PharmMapper, BATMAN-TCM, and ChEMBL) were used to explore the role of AST in disease prevention. Next, Veen map of AST targets and DEGs was used to explore the possible therapeutic targets for AST. Then we built the PPI network with the STRING database to identify the core genes. Docking analysis further verified that ferroptosis-related proteins (Hmox1, Myc, Nfe2l2, Sirt1, Jun, Map1lc3b, Tlr4, Keap1, and Tfrc) might be the potential targets for the prevention of PM_2.5_-induced cardiomyocyte injury with AST. Above results suggested that AST may attenuate PM_2.5_-induced cardiac injury by inhibiting ferroptosis.

There has been an increase in CVDs caused by ambient PM_2.5_ in recent years, and short-term exposure to PM_2.5_ has been related to a heightened cardiovascular risk [35]. However, the mechanism of the association between PM_2.5_ exposure and CVDs remains ambiguous and requires further study. AST is a xanthophyll carotenoid, and has been showed to have antioxidant and anti-inflammatory effects both in vitro and in vivo [36]. Previous studies have demonstrated that CVDs caused by PM_2.5_ exposure were accompanied by both activation of topical macrophages and inflammatory cell infiltration [37]. Inflammatory mediators play a significant role in cardiomyocyte injury induced by PM_2.5_ and reflect the severity of lesion damage to a certain extent. In this study, our results indicated that the levels of IL-6, TNF-a, and IL-1β in heart tissues and serum were increased by PM_2.5_ exposure but decreased by AST pretreatment. oxidative stress is also a mainly mechanism in the pathogenesis of PM_2.5_-induced cardiovascular injury [34]. PM_2.5_ can deposit in the alveolar region and enter the circulatory system via permeating alveolar epithelium and vascular endothelium [38]. This phenomenon might cause an imbalance between excessive generation of free radicals. As an antioxidant, AST contains conjugated double bonds, hydroxyl and keto groups that deliver electrons to free radicals and transform them into more stable products [39, 40]. Our finding points out that PM_2.5_ exposure could decrease the activities of GSH, SOD, and GSH-Px in the heart tissues and serum of the SD rats. In addition, after PM_2.5_ stimulation, the content of MDA substantially increased in the heart tissues and serum. However, pretreat with AST could alleviate these PM_2.5_-induced changes.

Research on the molecular mechanisms of ferroptosis and its potential applications has become increasingly popular since ferroptosis was reported in 2012 [41]. It is characterized by the accumulation of ROS and iron-dependent accumulation of lipid peroxidation [42]. In our study, excessive accumulation iron and the accumulation of ROS were concomitantly observed in PM_2.5_-treated rats and cardiomyocytes. Meanwhile, network pharmacology and bioinformatic analysis indicated that the ferroptosis signaling pathway was highly correlated with PM_2.5_-induced cardiomyocyte injury. Ferroptosis inhibitors Fer-1 showed an obvious effect against ferroptosis in both in vitro and in vivo models. These experimental results prove our conjecture that ferroptosis is engaged in the mechanism of PM_2.5_-induced cardiomyocyte injury. AST is a supernatural antioxidant that has been extensively studied [43, 44]. However, only very limited studies have reported the effect of AST on ferroptosis. A recent study noted that AST pretreatment could relieve LPS-induced lung injury, perhaps by suppressing ferroptosis [45]. In consistent with this study, our results explicitly defined the role of ferroptosis in cardiomyocyte injury caused by PM_2.5_. Our study revealed that AST pretreatment could counteract this effect by decreasing the levels of ROS and iron as effectively as Fer-1 in vitro and in vivo. To further investigate the mechanism of AST in preventing PM_2.5_-induced damage. We examined changes in the expression levels of iron metabolism-related proteins that were regulated by iron. The result showed that PM_2.5_ exposure elicited remarkable alterations in iron metabolism, leading to disturbances in iron homeostasis. However, AST alleviated these PM_2.5_-induced changes. GPX4 and SLC7A11 are considered as the characterizing factors of ferroptosis, and the downregulation of SLC7A11 and GPX4 expression is recognized as a reliable indicator of ferroptosis. Our results indicated that the expressions of SLC7A11 and GPX4 obviously decreased in the PM_2.5_ exposure group, AST pretreatment restored their expression. These results suggested that AST may act as an inhibitor of ferroptosis to alleviate myocardial injury induced by PM_2.5_.

The evidence obtained in this study may provide new insight into the AST in the prevention and treatment of PM_2.5_-induced CVDs. Our study also indicated that targeting ferroptosis signaling pathways may be an efficient route for alleviating PM_2.5_-induced cardiomyocyte injury. However, considering the limitations of the present study, the present results must be interpreted with caution. First, although intratracheal instillation is the commonly used model establishment method in toxicological studies of PM_2.5_, a real-world mixture of PM for exposure is a more meaningful measure to study preventative strategies. Second, the animals were pretreated with AST by oral gavage, which did not fully represent the requirements of AST-rich food by humans. Finally, our study only investigated that AST exerted cardioprotection via ferroptosis. Other forms of cell death are worth further investigation. The specific roles of AST in PM_2.5_-induced cardiomyocyte injury require further validation in future studies by more scientific and rigorous study design.

## Conclusion

The present study disclosed that AST could significantly protect the cardiomyocytes from oxidative stress and inflammation in vitro and in vivo. The molecular mechanism for the protective role of AST in the progress of cardiomyocyte injury induced by PM_2.5_ involves the inhibition of the ferroptosis signaling pathways. Therefore, AST, as a potential ferroptosis inhibitor, may offer a promising safe and effective approach for preventing PM_2.5_-induced cardiomyocyte injury.

### Supplementary Information

**Additional file 1: Supplementary Table S1. **Detailed information regarding the antibodies.

## Data Availability

The data that support the findings of this study are available on request from the corresponding author (Yuxia Ma), upon reasonable request.

## Abbreviations

CVDs: Cardiovascular diseases
AST: Astaxanthin
PM_2.5_: Fine particulate matter
PAHs: Polycyclic aromatic hydrocarbons
RCD: Regulatory cell death
ROS: Reactive oxygen species
Fer-1: Ferrostatin-1
DEGs: Differentially expressed genes
FRGs: Ferroptosis-related genes
CK: Creatine kinase
LDH: Lactate dehydrogenase
SOD: Superoxide dismutase
MDA: Malondialdehyde
GSH: Glutathione
CAT: Catalase
IL-6: Interleukin-6
FTH1: Ferritin heavy chain
FTL: Ferritin light chain
GPX4: Glutathione peroxidase 4
SLC7A11: Solute carrier family 7 member 11
TfR1: Transferrin receptor 1
