# Supplementary material for: Astaxanthin alleviates PM2.5-induced cardiomyocyte injury via inhibiting ferroptosis
Source: Cell Mol Biol Lett. 2023 Nov 25;28:95. doi: 10.1186/s11658-023-00513-1 (PMC10675963; doi:10.1186/s11658-023-00513-1)
Supplement: Supplementary file 1 — Additional file 1: Supplementary Table S1. Detailed information regarding the antibodies. [file 11658_2023_513_MOESM1_ESM.docx]

| Antibody name | Company | Cat# | Dilution ratio |
| --- | --- | --- | --- |
| TfR1 | ABclonal Technology (Wuhan, China) | #A5865 | 1:1000 |
| FTL | ABclonal Technology (Wuhan, China) | #A11241 | 1:1000 |
| FTH1 | ABclonal Technology (Wuhan, China) | #A19544 | 1:1000 |
| GPX4 | ABclonal Technology (Wuhan, China) | #A1933 | 1:1000 |
| SLC7A11 | ABclonal Technology (Wuhan, China) | #A2413 | 1:1000 |
| GAPDH | ABclonal Technology (Wuhan, China) | #A19056 | 1:1000 |
| Secondary antibody | Abways Technology (Shanghai, China) | #AB0101 | 1:20000 |

**Additional file Table S1**
